# Development and validation of a prediction model of length of ICU stay for benchmarking: a retrospective cohort study

**DOI:** 10.1038/s41598-026-56159-4

**Published:** 2026-06-03

**Authors:** Hideki Endo, Satoru Hashimoto

**Affiliations:** 1 Department of Healthcare Quality Assessment, Graduate School of Medicine, The University of Tokyo, 7-3-1 Hongo, Bunkyo-ku, Tokyo, 113-8655 Japan; 2 ICU Collaboration Network, 2-15-13 Hongo, Bunkyo-ku, Tokyo, 113-0033 Japan

**Keywords:** Efficiency, Length of intensive care unit stay, Prediction model, Benchmarking, Diseases, Health care, Medical research, Risk factors

## Abstract

Efficient use of intensive care unit (ICU) resources requires accurate prediction of length of stay (LOS), yet existing LOS models show inconsistent performance across settings and may not generalize to Japanese ICUs. We conducted a retrospective cohort study using the Japanese Intensive Care Patient Database, including adults admitted to 87 ICUs between April 2022 and March 2023. The primary outcome was ICU LOS in days. Generalized additive models with several distributional assumptions were fitted using the Acute Physiology and Chronic Health Evaluation III score, primary disease category, emergency surgery status, and admission source as predictors. Model performance was evaluated using deviance explained with internal validation. The best-performing model was applied to derive ICU-level standardized length-of-stay ratios (SLOSR) and observed minus expected length of stay (OMELOS), summarized using funnel plots with and without overdispersion correction. Among 65,395 patients, median ICU LOS was 3 days (interquartile range: 2–5). A gamma model with a log link achieved the highest cross-validated deviance explained (0.415). Overdispersion correction reduced the proportion of ICUs exceeding the 95% control limits from 69% to 9.2%. These findings indicate that a gamma-based generalized additive model enables case-mix–adjusted ICU LOS benchmarking and that overdispersion correction substantially affects outlier identification.

**Supplementary Information:**

The online version contains supplementary material available at 10.1038/s41598-026-56159-4.

## Introduction

Efficient use of healthcare resources is required owing to the inherently limited capacity. In the intensive care unit (ICU) setting, the length of ICU stay is an operational performance measure corresponding to efficiency and has been studied to optimize throughput^1–3^. Efficient care benefits patients by improving outcomes, controlling healthcare costs, and ensuring timely access to critical care. A key challenge in ICU management is to predict the length of stay (LOS) for individual patients accurately^4,5^. Prolonged or unexpected ICU stay can delay subsequent admissions, increase morbidity and mortality, and impose substantial financial burden^6–8^. However, previous studies that sought to predict ICU LOS displayed inconsistent performance across different populations and settings^4^. This variability may arise from differences in the case-mix, modeling approaches, and local healthcare practices, highlighting the limited generalizability of existing models. Because ICU organization and admission criteria vary considerably across countries, predictive models, especially those developed in Western healthcare systems, may not be directly applicable in Japan, where ICU bed availability, staffing ratios, and patient profiles differ. Because operational improvement is inherently local^9^, it is essential to assess whether established modeling approaches perform adequately in the Japanese context.

Therefore, our aim was to develop and validate a prediction model for ICU LOS using a multicenter Japanese clinical database, employing similar statistical methods to those used in previous studies. By evaluating model performance in this distinct healthcare environment, we sought to test whether existing modeling approaches are applicable or generalizable to Japanese ICUs.

## Results

**Fig. 1:**
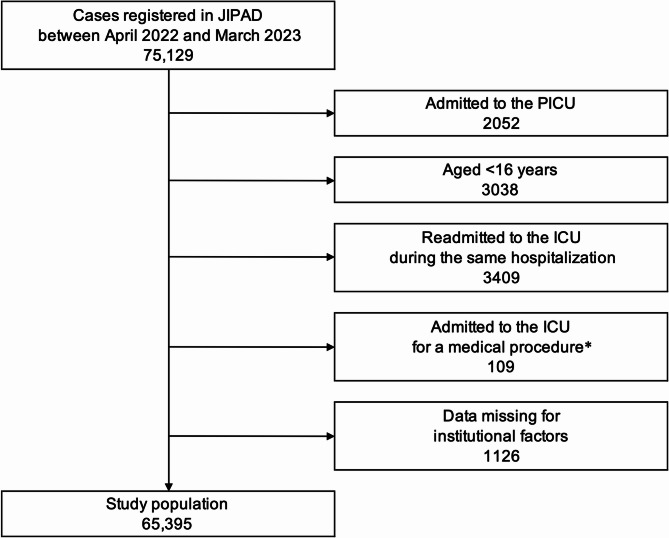
Study population flowchart. *ICU* intensive care unit, *PICU* pediatric intensive care unit. *Procedures such as central venous catheterization and cardioversion for atrial fibrillation.

After applying the exclusion criteria, the study population consisted of 65,395 patients admitted to 87 ICUs (Fig. 1). The number of patients with missing data for institutional factors was 1126, corresponding to the total number of admissions from two ICUs. Excluded admissions were less likely to be postoperative (lower proportion admitted from the operating room and elective surgery), whereas severity (APACHE III) and ICU length of stay were broadly similar between included and excluded admissions (Supplementary Table 1). The clinical characteristics of the study population is presented in Table 1. The median age was 71 years (interquartile range [IQR]: 59‒79 years), and 61.2% were male. About two-thirds of patients were admitted from the operating room, and emergency admissions comprised 45.6% of the total number of admissions. The top three admission diagnoses were cardiovascular (36.4%), gastrointestinal (19.0%), and neurological (14.1%). The median APACHE III score was 53 (IQR: 40‒72). The number of deaths recorded at ICU discharge was 2845 (4.4%). The median and mean ICU LOS were 3 days (IQR: 2‒5 days) and 4.5 days (standard deviation: 5.9 days), respectively. The characteristics of the ICUs are summarized in Supplementary Table 2. The median number of ICU beds was 12 (IQR: 8‒16) and the median number of intensivists was 4 (IQR: 3‒9). Approximately 70% of the ICUs were located in public hospitals.

**Table 1:** Clinical characteristics of the study population.

	Overall
Number of patients	65,395
Clinical characteristics	
Age (years), median (IQR)	71 (59, 79)
Male, *n* (%)	40,010 (61.2)
Admission source, *n* (%)	
Operating room	42,556 (65.1)
Emergency department	15,198 (23.2)
Ward	5311 (8.1)
Other types of care unit	1169 (1.8)
Other hospitals	1161 (1.8)
Emergency admission, *n* (%)	29,790 (45.6)
Admission type, *n* (%)	
Elective surgery	35,879 (54.9)
Emergency surgery	8432 (12.9)
Non-surgery	21,084 (32.2)
Admission diagnosis, *n* (%)	
Cardiovascular	23,810 (36.4)
Neurological	9224 (14.1)
Respiratory	8996 (13.8)
Gastrointestinal	12,406 (19.0)
Musculoskeletal	2049 (3.1)
Urogenital	2431 (3.7)
Trauma	1924 (2.9)
Sepsis	1265 (1.9)
Metabolic	1898 (2.9)
Hematologic	309 (0.5)
Gynecological	955 (1.5)
Other	128 (0.2)
Chronic illness, *n* (%)	
Immunosuppression	4098 (6.3)
Maintenance dialysis	3241 (5.0)
Metastatic tumor	2148 (3.3)
Respiratory failure	713 (1.1)
Liver cirrhosis	744 (1.1)
Heart failure	1229 (1.9)
Acute leukemia/multiple myeloma	398 (0.6)
Lymphoma	455 (0.7)
Liver failure	355 (0.5)
AIDS	37 (0.1)
Severity scores	
APACHE III score, median (IQR)	53 (40, 72)
APACHE III-j predicted mortality (%), median (IQR)	7.4 (2.9, 22.1)
APACHE III-j predicted mortality (%), mean (SD)	18.2 (24.1)
JROD predicted mortality (%), median (IQR)	1.7 (0.5, 7.2)
JROD predicted mortality (%), mean (SD)	9.0 (17.7)
Outcomes	
Length of ICU stay (days), median (IQR)	3 (2, 5)
Length of ICU stay (days), mean (SD)	4.5 (5.9)
Length of hospital stay (days), median (IQR)	19 (12, 34)
Length of hospital stay (days), mean (SD)	29.0 (38.3)
Number of deaths at ICU discharge (%)	2845 (4.4)
Number of deaths at hospital discharge (%)	5862 (9.0)

*AIDS* acquired immuno-deficiency syndrome, *APACHE* Acute Physiology and Chronic Health Evaluation, *ICU* intensive care unit, *IQR* interquartile range, *JROD* Japan Risk of Death, *SD* standard deviation.

The performance of each model is presented in Table 2. The generalized additive model with the gamma distribution and the log link function (model 4) had the highest cross-validated deviance explained (0.415; 95% CI: 0.415‒0.416) among the models without random intercepts. The RMSPE, MAPE, and prediction bias of model 4 were 5.370 (95% CI: 5.108‒5.619), 2.422 (95% CI: 2.368‒2.471), and 0.009 (95% CI: 0.005‒0.014), respectively. The *R*^2^ of model 4 at the ICU level was 0.408 (95% CI: 0.356‒0.449). Details of the final model specification, including the parametric coefficients and the smooth-term summary, are provided in Supplementary Tables 3 and 4.

**Table 2:** Model performance.

Model	Deviance explained (95% CI)	Cross-validated deviance explained (95% CI)	*R*^2^ at the patient level (95% CI)	RMSPE (95% CI)	MAPE (95% CI)	Prediction bias (95% CI)	*R*^2^ at the ICU level (95% CI)
1	0.188 (0.180 to 0.204)	0.183 (0.181 to 0.185)	0.188 (0.178 to 0.203)	5.356 (5.095 to 5.601)	2.436 (2.382 to 2.485)	0.007 (− 0.005 to 0.012)	0.404 (0.353 to 0.446)
2	0.340 (0.334 to 0.352)	0.333 (0.332 to 0.334)	0.187 (0.177 to 0.201)	5.360 (5.098 to 5.607)	2.423 (2.370 to 2.473)	0.000 (0.000 to 0.000)	0.408 (0.355 to 0.449)
3	0.408 (0.402 to 0.419)	0.401 (0.400 to 0.402)	0.185 (0.174 to 0.199)	5.366 (5.104 to 5.614)	2.423 (2.369 to 2.472)	0.007 (0.004 to 0.010)	0.408 (0.356 to 0.449)
4	0.422 (0.416 to 0.433)	0.415 (0.415 to 0.416)	0.184 (0.173 to 0.197)	5.370 (5.108 to 5.619)	2.422 (2.368 to 2.471)	0.009 (0.005 to 0.014)	0.408 (0.356 to 0.449)
5	0.420 (0.414 to 0.431)	0.414 (0.413 to 0.415)	0.183 (0.174 to 0.197)	5.375 (5.126 to 5.601)	2.410 (2.359 to 2.454)	−0.020 (− 0.040 to − 0.007)	0.397 (0.338 to 0.434)

Model 1: Generalized additive model with a Gaussian distribution and a log link function.

Model 2: Generalized additive model with a Poisson distribution and a log link function.

Model 3: Generalized additive model with a negative binomial distribution and a log link function.

Model 4: Generalized additive model with a gamma distribution and a log link function.

Model 5: Model 4 with random intercepts and random intercepts set to zero when calculating the length of stay.

*CI* confidence interval, *MAPE* mean absolute prediction error, *RMSPE* root mean square prediction error.

**Fig. 2:**
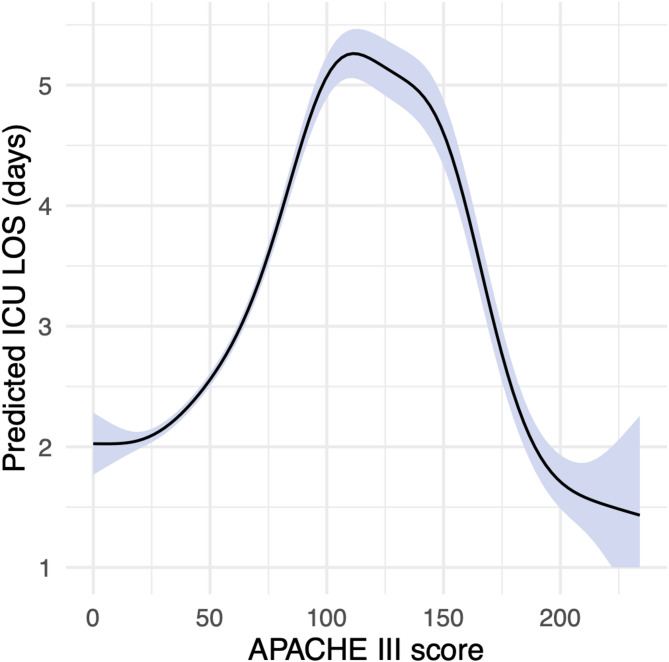
Association between APACHE III score and expected LOS. Band indicates the 95% CI. The primary disease code was set to gastrointestinal tumor with surgery, the emergency surgery status was non-emergent, and the source of admission was the operating room. *APACHE* Acute Physiology and Chronic Health Evaluation, *LOS* length of stay, *CI* confidence interval.

The association between the APACHE III score and predicted ICU LOS had an inverted U-shape (Fig. 2). Model 4 was chosen as the best model, and a random intercept term was added to account for clustering within the same ICU (model 5). The ICC of model 5 was 0.055. The cross-validated deviance explained was 0.414 (95% CI: 0.413‒0.415). Because the low ICC suggested no strong indication for including a random intercept and the deviance explained did not improve, model 4 was chosen as the best ICU LOS prediction model for benchmarking purposes.

**Fig. 3:**
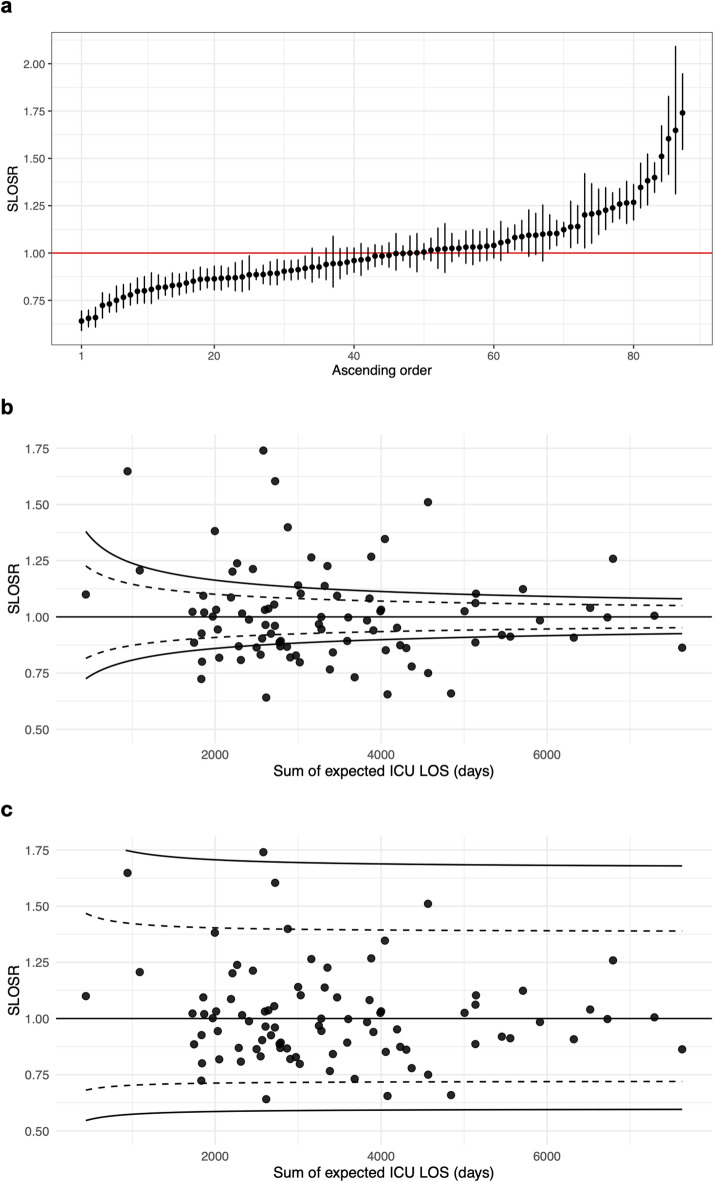
Plots for benchmarking with SLOSR. (**a**) Caterpillar plot, (**b**) Funnel plot without overdispersion correction, (**c**) Funnel plot with overdispersion correction. Error bars indicate the 95% confidence interval. Note that they only represent the uncertainty of the point estimates; they are not appropriate for detecting significantly different ICUs because of multiple testing. Dashed lines indicate the 95% control limits, and the solid lines the 99.8% control limits. *SLOSR* standardized length-of-stay ratio.

Figure 3 shows the caterpillar plot and the funnel plots of SLOSR using model 4. The SLOSR and OMELOS values with control limits are provided in Supplementary Table 5. The SLOSR ranged from 0.64 to 1.74. The funnel plot without overdispersion correction revealed that 60 ICUs (69%) exceeded the 95% control limits, and the plot with overdispersion correction showed that 8 ICUs (9.2%) performed beyond the 95% control limits. Seven ICUs (8.0%) were identified as outliers in SLOSR and OMELOS funnel plots with overdispersion correction. One ICU was an outlier only in the SLOSR plot, and two ICUs were outliers only in the OMELOS plot.

## Discussion

In this study, we developed and internally validated a prediction model for ICU LOS using a Japanese ICU database. A generalized additive model with a gamma distribution and a log link function showed the best performance. The ICU-level *R*^2^ was 0.408 (95% CI: 0.356–0.449), exceeding the previously suggested threshold of 0.36 for appropriate ICU comparability. The inverted U-shaped association between APACHE III score and ICU LOS was clearly demonstrated. The funnel plots of SLOSR and OMELOS without overdispersion correction showed high variability, in which over two-thirds of the ICUs were categorized as outliers. However, when applying overdispersion correction to the funnel plots, the number of outlier ICUs decreased markedly, meaning that the plots were plausible for benchmarking.

Modeling the association between severity scores and ICU LOS with a generalized additive model using a thin plate regression spline achieved good predictive performance. Verburg et al. used a generalized linear model with a restricted cubic spline, and the models fitted with a Poisson or Gaussian distribution showed the best performance^10^. Another study used a linear regression model with LOS truncated at 30 days^11^. The variability in the best models suggests that the model yielding the best performance is dependent on the population and setting. Therefore, each healthcare setting should test multiple models to determine which model best fits their environment. For comparability, we recommend that the explanatory variables include severity scores used in previous research, such as the variables in the APACHE scoring system^1,10^. Furthermore, the severity scores should be entered as a non-linear term in the regression model so that an inverted U-shape association or other complex associations can be captured.

Although our developed model revealed good applicability for benchmarking, the caterpillar and funnel plots displayed high variability among the ICUs. This suggests that ICUs operate differently beyond the differences in the case-mix, which was adjusted in the model when discharging patients from the ICU. Some ICUs with higher probability of discharging patients alive may require more time for the patients to recover from their severe conditions, or have a departmental policy for keeping the patient in the ICU for as long as possible. Because the reason cannot be determined from the funnel plot, the outlier ICUs warrant further investigation. Comparing SLOSR with SMR using a scatterplot may help researchers speculate about the reason for the longer ICU LOS^4^.

One of the strengths of this study is that we elucidated the inverted U-shape association between severity and ICU LOS. Previous studies used non-linear modeling but did not explicitly present this association^1,10^. An APACHE III score of 100 is given to patients with an extremely severe condition. Extremely high APACHE III scores reflect very severe illness with high mortality risk and shorter LOS. Although, the threshold of the APACHE III score may vary among healthcare settings, it is worth noting that extremely severe patients exceeding a certain risk threshold have a higher risk of death and hence a shorter ICU LOS.

A further strength is the evaluation of the model’s applicability to benchmarking using SLOSR and OMELOS. Because the rankings and outlier identification are scale-dependent, several benchmarking metrics should be used. The order of rankings differed between SLOSR and OMELOS, which is consistent with previous findings^5^. Our results provide additional evidence showing that rankings are not robust measures for performance assessment. However, seven ICUs (8.0%) were identified as outliers in SLOSR and OMELOS funnel plots with overdispersion correction. Because SLOSR is a relative measure and OMELOS is an absolute measure for quantifying differences in the mean ICU LOS, there may be some discrepancy in the detection of outliers between the two measures if the expected ICU LOS is very short or very long compared with the average value. However, if the SLOSR and OMELOS funnel plots locate an ICU in areas beyond the control limits, further investigation is needed to ascertain the causes of significant deviation from an average ICU.

Lastly, a strength of this study is that we evaluated the models using the deviance explained instead of the *R*^2^ value. In previous studies, model performance was often summarized using *R*^2^^4^, but this metric is generally interpreted in linear models with normally distributed errors. Because LOS is usually right-skewed, the deviance explained is a more appropriate metric for non-Gaussian models. We recommend using the deviance explained for evaluating model performance in future studies predicting the LOS. We have reported the *R*^2^ at the patient level along with the deviance explained for comparability with previous research. The *R*^2^ values were smaller than those of the deviance explained in all models except for the Gaussian model. This result indicates that *R*^2^ underestimates the fit of non-normally distributed models and thus should not be used.

A prediction model with good performance has major implications for operational improvement and management practices. The model was developed using data from the JIPAD. However, the JIPAD is not only a database but a project aimed at improving overall operational and clinical performance in the ICU. Currently, the JIPAD provides participating ICUs with an annual report comprising funnel plots of standardized mortality ratios. The mortality prediction model used to calculate the standardized mortality ratios is a recalibrated model of the APACHE III scoring system^12^. Because reliable performance measures are a key driver of local operational improvement^9^, ICU LOS benchmarking should be based on a well-calibrated and sufficiently predictive model tailored to the local setting. The ICU LOS prediction model developed in this study exhibited good predictive performance and thus can be used in funnel plots as an additional benchmarking tool for efficiency evaluation.

This study also has several limitations. First, the prediction model was developed using a Japanese ICU database and was not externally validated in other healthcare settings or future cohorts. Because the model was specifically designed for retrospective benchmarking to evaluate the relative performance of ICUs within the 2022 fiscal year, applying a fixed model formula to different regions or future years without modification is not appropriate. The effect of case-mix, institutional management, and operation of ICUs naturally evolves over time and varies across different countries. Therefore, when applying this framework to future years or other settings, annual recalibration will be necessary to establish a fair and contemporary baseline. Although temporal validation was not possible because data for the subsequent year were unavailable at the time of writing, future studies should evaluate the temporal transportability of the model alongside recalibration strategies. Second, because participation in the JIPAD is voluntary rather than mandatory, there is a potential selection bias. ICUs that are more proactive in clinical research, database registration, and performance improvement are more likely to participate. Therefore, the dataset may not perfectly represent the average standard of all ICUs nationwide in Japan, which should be considered when interpreting the generalizability of our findings. Third, we did not include operational characteristics that affect discharge of ICU patients in the regression model. Therefore, we could not ascertain why LOS varied among the ICUs. However, the operational characteristics are the target for improving efficiency. If we adjust for these characteristics, the LOS variations would not be detectable in the funnel plots. The specific ICU characteristics relevant to improving efficient use of ICUs remain to be identified in future studies. Finally, we did not stratify the model by survival status at ICU discharge because our model is intended for admission-time benchmarking using variables available at ICU admission. Exploring stratified analyses or dynamic prediction models that incorporate time-varying information during the ICU stay may be an important direction for future work.

In this nationwide multicenter cohort study, we developed an ICU LOS prediction model based on a generalized additive gamma model that exhibited adequate performance for case-mix-adjusted benchmarking. Applying the model to derive the OMELOS and SLOSR revealed substantial variations in ICU efficiency. The overdispersion-corrected funnel plots identified a limited number of ICUs with plausibly abnormal performance and demonstrated that the outlier classification depended on the chosen scale. The ICUs flagged as outliers by OMELOS and SLOSR may represent priority targets for in-depth review. External validation and incorporation of operational factors are important next steps.

## Methods

### Study design and setting

We performed a retrospective cohort study using the Japanese Intensive care PAtient Database (JIPAD), a multicenter registry of patients admitted to ICUs in Japan^13^. JIPAD started collecting data in 2015. As of April 2025, 155 ICUs were participating in JIPAD, and more than 552,000 admissions had been registered in the database. JIPAD collects baseline clinical characteristics at ICU admission, ICU procedures, and the patient’s outcome recorded in the ICU and at hospital discharge. The data are collected annually, registering patients admitted to the ICU between April 1 and March 31. This April–March cycle corresponds to the Japanese fiscal/administrative year, which is commonly used for annual reporting and data finalization in participating institutions and registries. The quality of data is checked during the initial phase when the ICU starts to collect data, and randomly thereafter. Further details are provided elsewhere^13^.

The Research Ethics Committee of the Faculty of Medicine of the University of Tokyo approved this study [approval number: 2022160NI-(2); approval date: July 18, 2024]. This study was conducted in accordance with the Declaration of Helsinki. Individual informed consent was waived because all data were analyzed in an anonymized fashion, but patients were given the opportunity to opt out. The Transparent Reporting of a multivariable prediction model for Individual Prognosis Or Diagnosis (TRIPOD) reporting statement was followed when preparing the manuscript^14^.

### Study population

The study population included patients who were admitted to an ICU between April 1, 2022, and March 31, 2023, and discharged by September 30, 2023. This period was chosen to use the most recent complete annual JIPAD dataset available at the time of analysis (defined on the fiscal-year basis described above) and to allow sufficient follow-up time for ICU discharge to be observed. Patients were excluded from the analysis if they were aged < 16 years, admitted to a pediatric ICU, readmitted to the ICU, admitted solely for performing a medical procedure such as central venous catheterization, or had missing values for the variables used in this analysis. The following variables were retrieved from the database to summarize the characteristics of the study population: age, sex, admission source, emergency admission, admission type, admission diagnosis, chronic illness, Acute Physiology and Chronic Health Evaluation (APACHE) III score, and Japan Risk of Death (JROD). Both the APACHE III and JROD scores were calculated based on data collected within the first 24 h of ICU admission. JROD is a recalibration of the APACHE III-j scoring system, which is adjusted to Japanese ICUs, for accurately predicting in-hospital mortality^12^.

### Outcomes

The primary outcome of interest was ICU LOS in whole days from ICU admission to discharge. Other outcomes that were only used to describe the characteristics of the study population included hospital LOS, ICU mortality, and hospital mortality.

### Statistical analysis

The model for predicting ICU LOS was developed with reference to previously published models^10^. Although previous models used linear or generalized linear models, we used a generalized additive model to incorporate flexible modeling^15^. We tested four models, which accounted for the following error terms: Gaussian, Poisson, negative binomial, and gamma distribution, all with a log link function^10^. Predictors included the APACHE III score, primary disease code, emergency surgery status, and source of admission. These predictors were chosen based on the APACHE III scoring system^16^. Predictors were pre-specified to align with established case-mix adjustment frameworks and to ensure feasibility using routinely available variables at ICU admission. The APACHE III score was included as a smooth term in the regression model. As the base function of the smooth term, we selected a thin plate regression spline, and applied the restricted maximum likelihood method to estimate the model parameters^15^.

Model performance was mainly evaluated using the proportion of deviance explained, which quantifies how much of the variability in ICU LOS is explained by the model^15^. This metric is conceptually similar to the coefficient of determination (*R*^2^), with values ranging from 0 to 1, where higher values indicate better model fit. Because the error terms are not normally distributed, *R*^2^ was calculated as a supplementary measure. To validate the developed model internally, we performed cross-validation of the proportion of deviance explained^17^. Ten-fold cross-validation was repeated 100 times, and the mean and the 2.5% and 97.5% percentiles of the deviance explained were calculated to present the optimism and uncertainty of the model performance. External validation was not performed because the purpose of developing the prediction model was to benchmark the performance of ICUs in the study period. Although ICU discharge can be conceptualized as a time-to-event process, our primary benchmarking metrics were based on the predicted mean ICU LOS in days. Therefore, we selected regression models for LOS with a log link as the primary modeling framework because they directly estimate the conditional mean LOS on the day scale, which aligns naturally with the calculation of ICU-level benchmarking measures. In contrast, time-to-event models primarily estimate hazards, and deriving a mean LOS requires additional modeling steps; restricted mean survival time also requires specification of a time horizon. We therefore did not adopt a time-to-event framework as the primary approach in this benchmarking-focused study. The model with the best fit was determined based on the cross-validated proportion of deviance explained. Other model performance metrics included the root mean square prediction error (RMSPE), the mean absolute prediction error (MAPE), and the prediction bias^10^. These measures are described in Supplementary Method 1. *R*^2^ at the ICU level was also calculated to assess the degree of comparability among ICUs. *R*^2^ of ≥ 0.36 was considered appropriate for comparing performance among ICUs^4^. Furthermore, because the ICU LOS for each patient was likely to be associated with that of other patients in the same ICU, an additional model was developed with a random intercept that accounted for ICU-level clustering, and the predictive performance was evaluated using the same dataset, except the random intercept was set to zero. The intraclass correlation coefficient (ICC) was calculated to evaluate the degree of correlation of ICU LOS in each ICU^18^. An ICC > 0.1 showed high patient-level correlation in the same ICU. To visualize the non-linear association between the APACHE III score and ICU LOS, the other variables in the regression model, namely, the primary disease code, emergency surgery status, and the source of admission, were fixed at the mode (i.e., the most frequently observed category).

To benchmark efficient use among the participating ICUs, we determined the standardized length-of-stay ratio (SLOSR) for each ICU. The SLOSR was defined as the ratio of the mean ICU LOS to the predicted ICU LOS^19^. The predicted ICU LOS was taken from the best-fit model described above. We also calculated the observed minus expected length of stay (OMELOS) to quantify the difference in the day-scale for managerial relevance. The OMELOS was added because benchmarking measures are scale-dependent^5^. OMELOS was defined as the mean observed ICU LOS minus the mean predicted ICU LOS in a particular ICU^20^. The formulae for SLOSR and OMELOS are given in Supplementary Method 2. The uncertainty for the ICU-level SLOSR and OMELOS was quantified by stratified nonparametric bootstrapping (1000 replicates); patients were resampled within the ICUs with replacement. Point estimates were computed for the original sample; 95% percentile confidence intervals (CIs) were derived from the bootstrap distribution. An average ICU is expected to have an OMELOS of 0 and a SLOSR of 1. Caterpillar plots were drawn to visualize the variations of ICU LOS among the ICUs, and funnel plots were built to detect outlier ICUs^21^. ICUs were classified as outliers if they fell outside the control limits of the funnel plots. Because benchmarking data in healthcare settings often show overdispersion (i.e., more between-ICU variability than assumed by nominal control limits), funnel plots with and without overdispersion correction were developed. We regarded this overdispersion primarily as a property of the ICU-level benchmarking stage, reflecting residual institutional and operational heterogeneity not captured by the patient-level case-mix model, rather than solely as evidence of patient-level model misspecification. ICU-level clustering was separately evaluated using a random-intercept model as described above. Overdispersion was corrected according to the methods proposed by Spiegelhalter^22^ to avoid spuriously identifying ICUs as outliers when observed between-ICU variation exceeded the nominal control limits.

*P*-values of ≤ 0.05 were considered statistically significant. All statistical analyses were performed using R version 4.4.1 (2024, R Foundation for Statistical Computing, Vienna, Austria). The *mgcv* package (version 1.9-3) was used to create the generalized additive models.

## Supplementary Information

Below is the link to the electronic supplementary material.

Supplementary Material 1

## Data Availability

The data that support the findings of this study are available from the Japanese Intensive Care PAtient Database (JIPAD), but restrictions apply to the availability of these data, which were used under license for the current study, and so are not publicly available. Data are however available from the authors upon reasonable request and with permission of the JIPAD Working Group.
