# Supplementary material for: Development and validation of a prediction model of length of ICU stay for benchmarking: a retrospective cohort study
Source: Sci Rep. 2026 Jun 3;16:25326. doi: 10.1038/s41598-026-56159-4 (PMC13473596; doi:10.1038/s41598-026-56159-4)
Supplement: Supplementary file 1 [file 41598_2026_56159_MOESM1_ESM.pdf]

Supplementary information

Title: **Development and validation of a prediction model of length of ICU stay for benchmarking: a retrospective cohort study**

Scientific Reports

Hideki Endo[1], Satoru Hashimoto[2]

[1]Department of Healthcare Quality Assessment, Graduate School of Medicine, The University of Tokyo, 7-3-1 Hongo, Bunkyo-ku, Tokyo 113-8655, Japan

[2]ICU Collaboration Network, 2-15-13 Hongo, Bunkyo-ku, Tokyo 113-0033, Japan

Corresponding author: Hideki Endo

E-mail: hidendo-thk@umin.ac.jp

Table of contents

Supplementary Method 1 Calculation of model performance measures 3

Supplementary Method 2 Calculation of SLOSR and OMELOS 4

Supplementary Table 1 Clinical characteristics of included vs excluded ICU admissions 5

Supplementary Table 2 Characteristics of the participating ICUs 6

Supplementary Table 3 Parametric coefficients for the selected model (Model 4) 7

Supplementary Table 4 Specification and summary of the smooth term in the selected model (Model 4). 11

Supplementary Table 5 SLOSR and OMELOS data for the individual ICUs 12

Supplementary Method 1 Calculation of model performance measures

Root mean square prediction error (RMSPE):

$$\sqrt{\frac{1}{n}\sum_{i=1}^{n}(\hat{y}_i - y_i)^2}$$

Mean absolute prediction error (MAPE):

$$\frac{1}{n}\sum_{i=1}^{n}|\hat{y}_i - y_i|$$

Prediction bias:

$$\frac{1}{n}\sum_{i=1}^{n}(\hat{y}_i - y_i)$$

Notation: n is the number of intensive care unit (ICU) patients, y_i is the observed length of stay (LOS) for patient *i,* and $\hat{y}_i$ is the predicted LOS for patient *i.*

Supplementary Method 2 Calculation of SLOSR and OMELOS

$$\bar{y}_j = \frac{1}{n_j} \sum_{i \in j} y_i$$

$$\bar{\hat{y}}_j = \frac{1}{n_j} \sum_{i \in j} \hat{y}_i$$

Standardized length-of-stay ratio (SLOSR)

$$\text{SLOSR}_j = \frac{\bar{y}_j}{\bar{\hat{y}}_j}$$

Observed minus expected length of stay (OMELOS)

$$\text{OMELOS}_j = \bar{y}_j - \bar{\hat{y}}_j$$

Notation: *n* is the number of ICU patients, y_i is the observed length of stay for patient *i*, $\hat{y}_i$ is the predicted length of stay for patient *i*, $\bar{y}_j$ is the average of observed length of stay for ICU *j*, and $\bar{\hat{y}}_j$ is the average predicted length of ICU stay for ICU *j*.

Supplementary Table 1 Clinical characteristics of included vs excluded ICU admissions

	Included	Excluded
Number of patients	65395	1126
Clinical characteristics		
Age (years), median (IQR)	71 (59, 79)	72 (60, 79)
Male, *n* (%)	40010 (61.2)	698 (62.0)
Admission source, *n* (%)		
Operating room	42556 (65.1)	540 (48.0)
Emergency department	15198 (23.2)	380 (33.7)
Ward	5311 (8.1)	146 (13.0)
Other types of care unit	1169 (1.8)	3 (0.3)
Other hospitals	1161 (1.8)	57 (5.1)
Admission type, *n* (%)		
Elective surgery	35879 (54.9)	454 (40.3)
Emergency surgery	8432 (12.9)	115 (10.2)
Non-surgery	21084 (32.2)	557 (49.5)
Severity scores		
APACHE III score, median (IQR)	53 (40, 72)	54 (39, 76)
Outcomes		
Length of ICU stay (days), median (IQR)	3 (2, 5)	3 (2, 5)
Length of ICU stay (days), mean (SD)	4.5 (5.9)	5.0 (7.3)
Length of hospital stay (days), median (IQR)	19 (12, 34)	19 (12, 35)
Length of hospital stay (days), mean (SD)	29.0 (38.3)	30.4 (36.0)
Number of deaths at ICU discharge (%)	2845 (4.4)	58 (5.2)
Number of deaths at hospital discharge (%)	5862 (9.0)	116 (10.3)

APACHE Acute Physiology and Chronic Health Evaluation, *ICU* intensive care unit, *IQR* interquartile range, *SD* standard deviation

Supplementary Table 2 Characteristics of the participating ICUs

	**Overall**
Number of ICUs	87
Number of ICU beds, median (IQR)	12 (8, 16)
Number of intensivists, median (IQR)	4 (3, 9)
Number of intensivists per bed, median (IQR)	0.40 (0.22, 0.69)
Number of hospital beds, median (IQR)	653 (543, 836)
Hospital to ICU bed ratio, median (IQR)	60.00 (47.06, 79.88)
Number of ICU admissions, median (IQR)	671 (469, 847)
Annual ICU admission to bed ratio, median (IQR)	63.92 (44.41, 76.12)
Type of hospital (%)	
Private non-university affiliated	13 (14.9)
Private university-affiliated	13 (14.9)
Public non-university affiliated	34 (39.1)
Public university-affiliated	27 (31.0)

ICU intensive care unit, *IQR* interquartile range.

Supplementary Table 3 Parametric coefficients for the selected model (Model 4)

Term	Estimate	Standard error	P value
Intercept	1.297	0.084	<0.001
Primary disease code 101	0.886	0.101	<0.001
Primary disease code 102	0.220	0.085	0.009
Primary disease code 103	0.380	0.087	<0.001
Primary disease code 104	0.114	0.084	0.17
Primary disease code 105	-0.025	0.124	0.84
Primary disease code 106	-0.063	0.096	0.51
Primary disease code 107	0.100	0.084	0.23
Primary disease code 108	-0.492	0.368	0.18
Primary disease code 109	0.146	0.090	0.10
Primary disease code 110	0.569	0.117	<0.001
Primary disease code 111	-0.134	0.100	0.18
Primary disease code 201	0.281	0.090	0.002
Primary disease code 202	0.064	0.138	0.64
Primary disease code 203	0.100	0.162	0.54
Primary disease code 204	0.628	0.107	<0.001
Primary disease code 206	0.176	0.117	0.13
Primary disease code 207	0.219	0.109	0.04
Primary disease code 208	0.202	0.099	0.04
Primary disease code 209	0.181	0.147	0.22
Primary disease code 210	0.766	0.161	<0.001
Primary disease code 211	0.544	0.088	<0.001
Primary disease code 212	0.388	0.090	<0.001
Primary disease code 213	0.478	0.086	<0.001
Primary disease code 301	0.635	0.106	<0.001
Primary disease code 303	-0.137	0.122	0.26
Primary disease code 305	-0.196	0.094	0.04
Primary disease code 306	-0.129	0.111	0.25
Primary disease code 307	-0.103	0.112	0.36
Primary disease code 308	0.247	0.117	0.03
Primary disease code 309	-0.136	0.126	0.28
Primary disease code 310	0.088	0.148	0.55
Primary disease code 311	0.471	0.107	<0.001
Primary disease code 312	0.345	0.125	0.006
Primary disease code 313	-0.084	0.093	0.37

Primary disease code 401	0.077	0.094	0.41
Primary disease code 402	0.498	0.099	<0.001
Primary disease code 403	-0.089	0.090	0.32
Primary disease code 404	0.686	0.130	<0.001
Primary disease code 405	-0.182	0.201	0.36
Primary disease code 406	0.918	0.133	<0.001
Primary disease code 407	0.211	0.090	0.02
Primary disease code 408	0.382	0.121	0.002
Primary disease code 409	0.035	0.143	0.80
Primary disease code 410	0.092	0.102	0.36
Primary disease code 501	0.148	0.106	0.16
Primary disease code 502	-0.140	0.118	0.24
Primary disease code 503	0.215	0.089	0.02
Primary disease code 504	-0.128	0.096	0.18
Primary disease code 601	0.321	0.091	<0.001
Primary disease code 602	0.173	0.092	0.06
Primary disease code 603	1.115	0.117	<0.001
Primary disease code 604	0.423	0.164	0.01
Primary disease code 605	0.509	0.155	0.001
Primary disease code 701	-0.131	0.104	0.21
Primary disease code 702	-0.183	0.096	0.06
Primary disease code 703	-0.126	0.092	0.17
Primary disease code 704	-0.127	0.088	0.15
Primary disease code 801	0.277	0.119	0.02
Primary disease code 802	0.277	0.102	0.007
Primary disease code 901	-0.046	0.097	0.64
Primary disease code 902	-0.366	0.368	0.32
Primary disease code 903	-0.438	0.128	<0.001
Primary disease code 1101	0.408	0.123	<0.001
Primary disease code 1102	0.192	0.104	0.06
Primary disease code 1202	-0.407	0.098	<0.001
Primary disease code 1203	-0.293	0.102	0.004
Primary disease code 1204	0.259	0.086	0.003
Primary disease code 1205	-0.206	0.107	0.05
Primary disease code 1206	0.254	0.086	0.003
Primary disease code 1207	0.239	0.087	0.006
Primary disease code 1208	-0.086	0.086	0.32

Primary disease code 1209	0.319	0.090	<0.001
Primary disease code 1210	0.063	0.098	0.52
Primary disease code 1211	-0.084	0.175	0.63
Primary disease code 1212	0.467	0.093	<0.001
Primary disease code 1213	-0.303	0.088	<0.001
Primary disease code 1214	-0.316	0.098	0.001
Primary disease code 1301	-0.065	0.113	0.56
Primary disease code 1302	-0.360	0.086	<0.001
Primary disease code 1303	-0.161	0.088	0.07
Primary disease code 1304	0.089	0.093	0.34
Primary disease code 1401	-0.248	0.091	0.006
Primary disease code 1403	-0.295	0.134	0.03
Primary disease code 1404	-0.448	0.094	<0.001
Primary disease code 1405	-0.244	0.085	0.004
Primary disease code 1406	-0.482	0.103	<0.001
Primary disease code 1407	0.884	0.129	<0.001
Primary disease code 1408	-0.286	0.093	0.002
Primary disease code 1409	-0.231	0.137	0.09
Primary disease code 1410	-0.196	0.109	0.07
Primary disease code 1411	-0.261	0.278	0.35
Primary disease code 1412	-0.340	0.115	0.003
Primary disease code 1413	-0.327	0.112	0.004
Primary disease code 1501	-0.043	0.095	0.65
Primary disease code 1502	-0.271	0.104	0.009
Primary disease code 1503	0.372	0.092	<0.001
Primary disease code 1504	-0.292	0.088	<0.001
Primary disease code 1505	-0.241	0.087	0.005
Primary disease code 1506	-0.222	0.090	0.01
Primary disease code 1507	-0.212	0.099	0.03
Primary disease code 1508	-0.334	0.090	<0.001
Primary disease code 1509	-0.411	0.100	<0.001
Primary disease code 1601	0.145	0.100	0.15
Primary disease code 1602	-0.081	0.096	0.40
Primary disease code 1603	0.639	0.250	0.01
Primary disease code 1604	0.257	0.160	0.11
Primary disease code 1605	-0.018	0.159	0.91
Primary disease code 1701	-0.394	0.087	<0.001

Primary disease code 1703	-0.435	0.102	<0.001
Primary disease code 1704	-0.038	0.101	0.71
Primary disease code 1705	-0.292	0.250	0.24
Primary disease code 1801	-0.352	0.092	<0.001
Primary disease code 1802	-0.506	0.108	<0.001
Primary disease code 1803	-0.348	0.105	<0.001
Primary disease code 1902	-0.351	0.089	<0.001
Primary disease code 1903	-0.150	0.093	0.11
Primary disease code 1904	0.012	0.105	0.91
Primary disease code 2101	-0.416	0.268	0.12
Primary disease code 2201	-0.264	0.100	0.008
Emergency surgery	0.398	0.018	<0.001
Admission source: emergency department	0.051	0.023	0.03
Admission source: ward	0.201	0.025	<0.001
Admission source: other types of care unit	0.344	0.034	<0.001
Admission source: other hospitals	0.370	0.034	<0.001

Supplementary Table 4 Specification and summary of the smooth term in the selected model (Model 4)

Smooth term	APACHE III score
Basis	Thin plate regression spline
Family (link)	Gamma (log)
Smoothing method	REML
edf	8.323
Ref.df	8.778
Test statistic	F = 483.1
p-value	<0.001

Note: edf indicates the effective degrees of freedom of the smooth term (reflecting its complexity); Ref.df indicates the reference degrees of freedom used for the approximate F-test.

Supplementary Table 5 SLOSR and OMELOS data for the individual ICUs

ICU ID	Ascending order by SLOSR	SLOSR	Flag without overdispersion correction	Flag with overdispersion correction	Ascending order by OMELOS	OMELOS	Flag without overdispersion correction	Flag with overdispersion correction
ICU1	1	0.641	Beyond 99.8% control limit	Beyond 95% control limit	3	–2.001	Beyond 99.8% control limit	Beyond 95% control limit
ICU2	2	0.655	Beyond 99.8% control limit	Beyond 95% control limit	2	–2.095	Beyond 99.8% control limit	Beyond 95% control limit
ICU3	3	0.659	Beyond 99.8% control limit	Beyond 95% control limit	1	–2.692	Beyond 99.8% control limit	Beyond 95% control limit
ICU4	4	0.724	Beyond 99.8% control limit	Within control limit	4	–1.614	Beyond 99.8% control limit	Within control limit
ICU5	5	0.731	Beyond 99.8% control limit	Within control limit	10	–1.167	Beyond 99.8% control limit	Within control limit
ICU6	6	0.750	Beyond 99.8% control limit	Within control limit	5	–1.470	Beyond 99.8% control limit	Within control limit
ICU7	7	0.766	Beyond 99.8% control limit	Within control limit	7	–1.395	Beyond 99.8% control limit	Within control limit
ICU8	8	0.779	Beyond 99.8% control limit	Within control limit	8	–1.338	Beyond 99.8% control limit	Within control limit
ICU9	9	0.798	Beyond 99.8% control limit	Within control limit	6	–1.453	Beyond 99.8% control limit	Within control limit
ICU10	10	0.801	Beyond 99.8% control limit	Within control limit	11	–1.040	Beyond 99.8% control limit	Within control limit
ICU11	11	0.808	Beyond 99.8% control limit	Within control limit	9	–1.323	Beyond 99.8% control limit	Within control limit
ICU12	12	0.818	Beyond 99.8% control limit	Within control limit	12	–0.889	Beyond 99.8% control limit	Within control limit
ICU13	13	0.820	Beyond 99.8% control limit	Within control limit	19	–0.654	Beyond 99.8% control limit	Within control limit
ICU14	14	0.828	Beyond 99.8% control limit	Within control limit	15	–0.794	Beyond 99.8% control limit	Within control limit
ICU15	15	0.832	Beyond 99.8% control limit	Within control limit	21	–0.644	Beyond 99.8% control limit	Within control limit
ICU16	16	0.842	Beyond 99.8% control limit	Within control limit	14	–0.881	Beyond 99.8% control limit	Within control limit
ICU17	17	0.851	Beyond 99.8% control limit	Within control limit	13	–0.887	Beyond 99.8% control limit	Within control limit

ICU18	18	0.861	Beyond 99.8% control limit	Within control limit	20	–0.647	Beyond 99.8% control limit	Within control limit
ICU19	19	0.863	Beyond 99.8% control limit	Within control limit	16	–0.698	Beyond 99.8% control limit	Within control limit
ICU20	20	0.864	Beyond 99.8% control limit	Within control limit	23	–0.610	Beyond 95% control limit	Within control limit
ICU21	21	0.867	Beyond 99.8% control limit	Within control limit	24	–0.595	Beyond 99.8% control limit	Within control limit
ICU22	22	0.869	Beyond 99.8% control limit	Within control limit	27	–0.497	Beyond 99.8% control limit	Within control limit
ICU23	23	0.869	Beyond 95% control limit	Within control limit	18	–0.680	Beyond 95% control limit	Within control limit
ICU24	24	0.874	Beyond 99.8% control limit	Within control limit	17	–0.696	Beyond 99.8% control limit	Within control limit
ICU25	25	0.885	Beyond 95% control limit	Within control limit	25	–0.543	Beyond 95% control limit	Within control limit
ICU26	26	0.886	Beyond 99.8% control limit	Within control limit	32	–0.355	Beyond 99.8% control limit	Within control limit
ICU27	27	0.886	Beyond 99.8% control limit	Within control limit	28	–0.422	Beyond 95% control limit	Within control limit
ICU28	28	0.893	Beyond 95% control limit	Within control limit	26	–0.523	Beyond 95% control limit	Within control limit
ICU29	29	0.893	Beyond 95% control limit	Within control limit	22	–0.639	Beyond 95% control limit	Within control limit
ICU30	30	0.904	Beyond 95% control limit	Within control limit	34	–0.308	Beyond 95% control limit	Within control limit
ICU31	31	0.908	Beyond 99.8% control limit	Within control limit	29	–0.414	Beyond 99.8% control limit	Within control limit
ICU32	32	0.912	Beyond 99.8% control limit	Within control limit	31	–0.382	Beyond 95% control limit	Within control limit
ICU33	33	0.919	Beyond 95% control limit	Within control limit	35	–0.305	Beyond 95% control limit	Within control limit
ICU34	34	0.925	Beyond 95% control limit	Within control limit	36	–0.304	Within control limit	Within control limit
ICU35	35	0.926	Within control limit	Within control limit	37	–0.280	Within control limit	Within control limit
ICU36	36	0.940	Within control limit	Within control limit	30	–0.399	Within control limit	Within control limit

ICU37	37	0.944	Within control limit	Within control limit	33	–0.324	Within control limit	Within control limit
ICU38	38	0.945	Within control limit	Within control limit	38	–0.224	Within control limit	Within control limit
ICU39	39	0.952	Within control limit	Within control limit	39	–0.202	Within control limit	Within control limit
ICU40	40	0.960	Within control limit	Within control limit	40	–0.185	Within control limit	Within control limit
ICU41	41	0.964	Within control limit	Within control limit	41	–0.160	Within control limit	Within control limit
ICU42	42	0.968	Within control limit	Within control limit	42	–0.123	Within control limit	Within control limit
ICU43	43	0.984	Within control limit	Within control limit	43	–0.082	Within control limit	Within control limit
ICU44	44	0.984	Within control limit	Within control limit	44	–0.059	Within control limit	Within control limit
ICU45	45	0.988	Within control limit	Within control limit	45	–0.043	Within control limit	Within control limit
ICU46	46	0.997	Within control limit	Within control limit	46	–0.016	Within control limit	Within control limit
ICU47	47	0.998	Within control limit	Within control limit	47	–0.010	Within control limit	Within control limit
ICU48	48	1.000	Within control limit	Within control limit	48	–0.001	Within control limit	Within control limit
ICU49	49	1.001	Within control limit	Within control limit	49	0.007	Within control limit	Within control limit
ICU50	50	1.005	Within control limit	Within control limit	50	0.018	Within control limit	Within control limit
ICU51	51	1.015	Within control limit	Within control limit	51	0.050	Within control limit	Within control limit
ICU52	52	1.020	Within control limit	Within control limit	52	0.088	Within control limit	Within control limit
ICU53	53	1.022	Within control limit	Within control limit	53	0.091	Within control limit	Within control limit
ICU54	54	1.025	Within control limit	Within control limit	56	0.130	Within control limit	Within control limit
ICU55	55	1.025	Within control limit	Within control limit	55	0.125	Within control limit	Within control limit

ICU56	56	1.031	Within control limit	Within control limit	59	0.192	Within control limit	Within control limit
ICU57	57	1.032	Within control limit	Within control limit	60	0.209	Within control limit	Within control limit
ICU58	58	1.032	Within control limit	Within control limit	54	0.101	Within control limit	Within control limit
ICU59	59	1.036	Within control limit	Within control limit	58	0.180	Within control limit	Within control limit
ICU60	60	1.040	Within control limit	Within control limit	57	0.161	Within control limit	Within control limit
ICU61	61	1.055	Within control limit	Within control limit	62	0.303	Within control limit	Within control limit
ICU62	62	1.061	Beyond 95% control limit	Within control limit	61	0.217	Beyond 95% control limit	Within control limit
ICU63	63	1.082	Beyond 95% control limit	Within control limit	64	0.374	Beyond 95% control limit	Within control limit
ICU64	64	1.087	Within control limit	Within control limit	69	0.481	Within control limit	Within control limit
ICU65	65	1.094	Beyond 95% control limit	Within control limit	66	0.436	Beyond 95% control limit	Within control limit
ICU66	66	1.094	Within control limit	Within control limit	67	0.446	Within control limit	Within control limit
ICU67	67	1.100	Within control limit	Within control limit	63	0.314	Within control limit	Within control limit
ICU68	68	1.103	Beyond 99.8% control limit	Within control limit	65	0.432	Beyond 99.8% control limit	Within control limit
ICU69	69	1.104	Beyond 95% control limit	Within control limit	70	0.596	Beyond 95% control limit	Within control limit
ICU70	70	1.124	Beyond 99.8% control limit	Within control limit	68	0.449	Beyond 99.8% control limit	Within control limit
ICU71	71	1.138	Beyond 95% control limit	Within control limit	73	0.800	Beyond 99.8% control limit	Within control limit
ICU72	72	1.140	Beyond 99.8% control limit	Within control limit	71	0.625	Beyond 99.8% control limit	Within control limit
ICU73	73	1.201	Beyond 99.8% control limit	Within control limit	76	1.034	Beyond 99.8% control limit	Within control limit
ICU74	74	1.207	Beyond 95% control limit	Within control limit	78	1.205	Beyond 95% control limit	Within control limit

ICU75	75	1.213	Beyond 99.8% control limit	Within control limit	77	1.177	Beyond 99.8% control limit	Within control limit
ICU76	76	1.226	Beyond 99.8% control limit	Within control limit	72	0.718	Beyond 99.8% control limit	Within control limit
ICU77	77	1.238	Beyond 99.8% control limit	Within control limit	75	0.892	Beyond 99.8% control limit	Within control limit
ICU78	78	1.259	Beyond 99.8% control limit	Within control limit	79	1.228	Beyond 99.8% control limit	Within control limit
ICU79	79	1.265	Beyond 99.8% control limit	Within control limit	80	1.229	Beyond 99.8% control limit	Within control limit
ICU80	80	1.268	Beyond 99.8% control limit	Within control limit	74	0.820	Beyond 99.8% control limit	Within control limit
ICU81	81	1.347	Beyond 99.8% control limit	Within control limit	82	1.741	Beyond 99.8% control limit	Beyond 95% control limit
ICU82	82	1.382	Beyond 99.8% control limit	Within control limit	84	2.601	Beyond 99.8% control limit	Beyond 95% control limit
ICU83	83	1.399	Beyond 99.8% control limit	Beyond 95% control limit	81	1.570	Beyond 99.8% control limit	Within control limit
ICU84	84	1.510	Beyond 99.8% control limit	Beyond 95% control limit	83	2.208	Beyond 99.8% control limit	Beyond 95% control limit
ICU85	85	1.604	Beyond 99.8% control limit	Beyond 95% control limit	85	3.249	Beyond 99.8% control limit	Beyond 99.8% control limit
ICU86	86	1.648	Beyond 99.8% control limit	Beyond 95% control limit	87	4.305	Beyond 99.8% control limit	Beyond 99.8% control limit
ICU87	87	1.740	Beyond 99.8% control limit	Beyond 99.8% control limit	86	3.638	Beyond 99.8% control limit	Beyond 99.8% control limit

OMELOS observed minus expected length of stay, *SLOSR* standardized length-of-stay ratio
